# The Precision HYpertenSIon Care (PHYSIC) study: a double-blind, randomized, repeated cross-over study

**DOI:** 10.1080/03009734.2018.1498958

**Published:** 2018-09-28

**Authors:** Johan Sundström, Lars Lind, Shamim Nowrouzi, Per Lytsy, Kerstin Marttala, Inger Ekman, Patrik Öhagen, Ollie Östlund

**Affiliations:** a Department of Medical Sciences, Uppsala University, Uppsala, Sweden;; b Uppsala Clinical Research Center (UCR), Uppsala, Sweden

**Keywords:** Hypertension; blood pressure; randomised clinical trial; precision medicine

## Abstract

High blood pressure is the leading risk factor for premature deaths and a major cost to societies worldwide. Effective blood pressure-lowering drugs are available, but patient adherence to them is low, likely partly due to side effects. To identify patient-specific differences in treatment effects, a repeated cross-over design, where the same treatment contrasts are repeated within each patient, is needed. Such designs have been surprisingly rarely used, given the current focus on precision medicine. The Precision HYpertenSIon Care (PHYSIC) study aims to investigate if there is a consistent between-person variation in blood pressure response to the common blood pressure-lowering drug classes of a clinically relevant magnitude, given the within-person variation in blood pressure. The study will also investigate the between-person variation in side effects of the drugs. In a double-blind, randomized, repeated cross-over trial, 300 patients with mild hypertension will be treated with four blood pressure-lowering drugs (candesartan, lisinopril, amlodipine, and hydrochlorothiazide) in monotherapy, with two of the drugs repeated for each patient. If the study indicates that there is a potential for precision hypertension care, the most promising predictors of blood pressure and side effect response to the drugs will be explored, as will the potential for development of a biomarker panel to rank the suitability of blood pressure-lowering drug classes for individual patients in terms of anticipated blood pressure effects and side effects, with the ultimate goal to maximize adherence. The study follows a protocol pre-registered at ClinicalTrials.gov with the identifier NCT02774460.

## Background

High blood pressure is the leading risk factor for premature deaths worldwide (1) and accounts for more than 10% of all health care costs in some developed countries, with a mere fifth of that attributable to medication costs, and four-fifths to complications (2). Less than half of those who have high blood pressure are aware of it, and, of those receiving blood pressure-lowering drug treatment, only one-third have their blood pressure at target levels (3).

Effective blood pressure-lowering drugs are available, with the first-line drug classes—angiotensin-converting enzyme (ACE)-inhibitors, angiotensin receptor blockers, calcium channel blockers, and thiazide diuretics—considered equally effective on average. Most of their protective effects depend on the amount of blood pressure reduction achieved, and there are few class-specific protective effects (4). They do, however, have class-specific side effects.

Poor adherence is likely the main cause of poor blood pressure control, with as little as 1 in 2 patients estimated to be adherent to prescribed treatment in developed countries (5). Because poorly controlled blood pressure is such an important burden on patients and health care systems, improving adherence is imperative.

The concept of precision medicine promises maximization of treatment benefit and minimization of treatment harm using subtle characteristics (typically involving, but not limited to, gene variants) of patients with the same trait to guide treatment decisions. If the promise holds true widely, hypertension would be an optimal field, with a handful of first-line drug classes that all have similar average blood pressure-lowering effects, and each has common side effects that hamper adherence.

For hypertension, the concept needs answers to several important questions. The most important question is if there is a clinically relevant and repeatable between-person difference in blood pressure response or side effects to a given drug (6). If there is not, then there is no potential for precision medicine. Analysing variance components in parallel group trials (which cannot disentangle all of the relevant variance components) (7), a clinically irrelevant between-person difference in blood pressure response has been suggested to be the scenario for ACE-inhibitors (8), but this is unconfirmed and unknown for other drug classes. It is also unknown if between-person differences in blood pressure response are different for different drugs (9,10), and magnitudes of within- and between-person variations in side effects are particularly unknown. Resolving these issues is imperative for the possibilities of precision blood pressure-lowering treatment, and the present study, the Precision HYpertenSIon Care (PHYSIC) study, aims to answer those questions. Proper methods for that purpose have been proposed (7,11) and are used in this study.

If this study indicates that there is a potential for personalized blood pressure-lowering treatment, we will explore the most promising predictors of blood pressure and side effect response to the drugs, using peripheral blood analyses in combination with clinical data. If feasible, we will then explore the potential for development of a biomarker panel to rank the suitability of each of the blood pressure-lowering drug classes for the individual patient in terms of both anticipated blood pressure effect and side effects, with the ultimate goal to maximize adherence.

## Objectives

### Primary objective

The primary objective is to establish the potential for precision hypertension care, by investigating if there is a consistent between-person variation in blood pressure response to the common blood pressure-lowering drug classes of a clinically relevant magnitude, given the within-person variation in blood pressure.

### Secondary objectives

- To investigate the between-person variation in side effects of blood pressure-lowering drugs in contrast to the within-person variation in side effects.
- To investigate patient treatment preferences when presented with: i) their individual ratings for each drug class; ii) a combination of their individual ratings and blood pressure effect of each drug class.
- To develop a prediction model based on biomarkers and clinical characteristics for patient treatment preferences.
- To develop and evaluate a point-of-care tool using this prediction model, in order to inform treatment decisions in the clinical setting.

### Tertiary objectives

- To evaluate between- and within-person variation in response in mean and standard deviation of daytime, nighttime, and 24-h blood pressure and heart rate to the common blood pressure-lowering drug classes.
- To evaluate between- and within-person variation in self-reported symptoms and quality of life in response to the common blood pressure-lowering drug classes.
- To evaluate between- and within-person variation in pulse wave characteristics and estimated central blood pressure in response to the common blood pressure-lowering drug classes.
- To evaluate between- and within-person variation in DNA methylation, RNA, proteins, and metabolites in response to the common blood pressure-lowering drug classes.
- To investigate associations of DNA genomic variation, DNA methylation, RNA, proteins, and metabolites with blood pressure effects and self-reported symptoms and quality of life.
- To investigate the associations of self-reported symptoms and quality of life collected using an electronic diary with patients’ treatment preferences and adverse events reported in the clinic.

## Study design

### Rationale

To identify the patient-by-treatment interactions and hence investigate the patient-specific differences in treatment effects, a repeated cross-over design, where the same treatment contrasts are repeated within each patient, is needed. This has been convincingly suggested for decades (12), but such designs have been surprisingly rarely used given the current focus on precision medicine. In the PHYSIC trial, each patient is given all four first-line hypertension drugs, one at a time, and in addition repeats two of the treatments. This allows us to quantify whether there is consistent preference of one treatment over another within a blinded patient over different treatment periods. Separation of period effects and drug effects is key. Without pairwise repetition of treatments, a trial cannot assess whether a patient that preferred a certain treatment over another did so because of the treatment or because of an external factor such as the season or normal day-to-day variation. It would also have been impossible to assess whether a variation in treatment effects was due to differences between drugs in on-treatment within-patient variation, such as patients having larger day-to-day or season-to-season variations when on one of the drugs.

The study needs to be double-blind, and the order of the treatment periods randomized, in order to avoid obvious sources of bias.

Evidence-based titration and target doses have been chosen for all four drug classes included in this protocol (13). Within each drug class a compound that fitted the gelatin capsules was chosen. The suggested treatment duration per treatment arm is based on recent guidelines (14).

**Figure 1.**
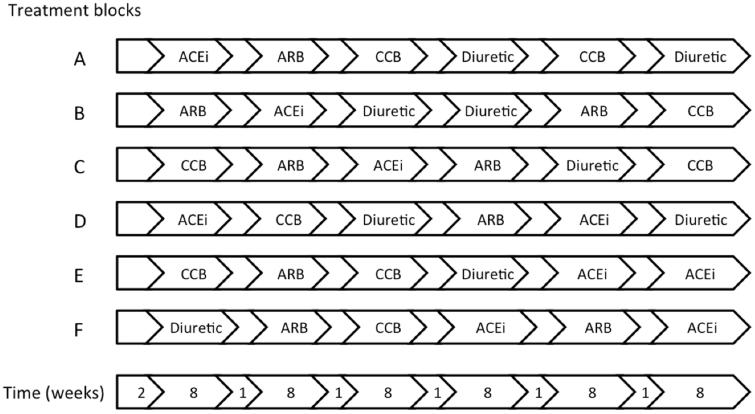
Study design. Each patient is randomly assigned to a treatment sequence created from one of the six possible combinations, by random permutation of the order in which the active treatments are given (the figure is a mere illustration of a few examples of possible sequences). Hence, 1/6 of the patients receive ACEi and ARB in two periods each, 1/6 of patients repeat the ACEi and CCB periods, 1/6 of the patients repeat the ACEi and Diuretic periods, 1/6 repeat the ARB and CCB periods, 1/6 repeat the ARB and Diuretic periods, and 1/6 repeat the CCB and Diuretic periods. Block randomization is used to ensure that approximately the same number of patients are assigned to each of the six combinations of active treatments. ACEi = angiotensin-converting enzyme inhibitor; ARB = angiotensin receptor blocker; CCB = calcium channel blocker; Diuretic = thiazide diuretic. Blank arrows indicate placebo run-in and wash-out.

The study is outlined in Table 1, examples of treatment sequences illustrated in Figure 1, and the sequence of events described in Table 2.

**Table 1.** Study outline.

Setting	Patients diagnosed with hypertension with a systolic blood pressure between 140 and 159 mmHg within five years before the start of the trial. Patients must be pharmacologically untreated or use blood pressure-lowering monotherapy at the time of recruitment to the present study.
Design	Double-blind, randomized, repeated cross-over, single-centre study.
Number of patients (planned)	300 patients included to obtain 240 evaluable patients.
Interventions	Candesartan: 8 mg weeks 1–2; 16 mg weeks 3–8 Lisinopril: 10 mg weeks 1–2; 20 mg weeks 3–8 Amlodipine: 5 mg weeks 1–2; 10 mg weeks 3–8 Hydrochlorothiazide: 12.5 mg weeks 1–2; 25 mg weeks 3–8.
Treatment duration	The study consists of six active treatment periods: four classes of blood pressure-lowering drugs will be tested, treatment with two classes are repeated for each patient. Patients will be assigned a treatment order based on block randomization ensuring that the same number of patients will repeat each treatment. Active drug is given during 6–8 weeks of each treatment period (titration dose week 1–2, target dose week 3–8). Placebo will be administered during 2 weeks of placebo run-in (between visit 1 and 2) and during 7 days’ wash-out between treatment periods. This gives a total duration of treatment of 47–59 weeks for each included patient.
Outcome	The primary analysis of individual variation in treatment differences in visit 4 daytime (10.00–20.00) ambulatory systolic blood pressure will be performed using a random effects model with correlated random intercept and treatment contrasts, allowing correlation between all random effects, and with treatment period as a fixed effect. All available periods with >90% treatment adherence from all randomized patients will be used.

**Table 2.** Sequence of events.

Visit	1	Placebo run-in (≥2 weeks)	2	Treatment period			5
				Dosing at home	3	4	
Week	–1 –(–2)		1	1–7	7	8	
Day	–7 –(–14)		1	2–54	55	56	
Informed consent	✓						
Inclusion/exclusion criteria	✓		✓				
Anthropometry^a^	✓						
Physical examination	✓						
Office BP^b^	✓		✓		✓		
Concomitant medication	✓		✓		✓		
Safety samples^c^	✓		✓		✓^d^		
DNA genomic samples^e^	✓						
Medical history Q^g^	✓						
Beliefs about medicine Q	✓						
Placebo dosing		✓					✓
Randomization			✓				
Study drug dosing^h^			✓	✓	✓	✓	
Staff train patients in electronic diary^f^			✓				
Self-reporting of symptoms in electronic diary^i^				✓	✓		
PERSYVE Q			✓		✓		
Pulse wave analysis			✓				
Capillary blood sampling and dried blood spots			✓				
Venous blood samples for DNA methylation, RNA, other biomarkers^j^			✓		✓		
AE/SAE recording^k^			✓		✓^q^		
Treatment preference rating Q^l^					✓		
24-h BP/ECG^m^			✓^n^		✓	✓	
Return of unused study drug by patients					✓		
Supply next study drug and 7-day placebo					✓		
Overall treatment preference rating^o^							✓
Prescription of preferred drug for continuous use^o^							✓

^a^ Measured using standard field protocols.

^b^ Prior to measurement patient will rest sitting for 10 min. Measurement twice within 5 min in-between measurements using a sphygmomanometer.

^c^ The following safety samples will be analysed: Hb, HbA1c, sodium, potassium, calcium, creatinine, ASAT, ALAT, γ-GT, and pregnancy test.

^d^ Last safety samples are taken and analysed at visit 3, in the last treatment period only.

^e^ Whole blood sample taken in two 7/6 ml EDTA Vacutainers^®^.

^f^ Patient fills in the electronic diary (PERSYVE Q) under supervision of study staff.

^g^ Q covering demographic items, medical history, smoking, and physical activity.

^h^ Dosing once daily in all treatment periods.

^i^ When patients are dosing at home (between visit 2 and 3) symptoms will be recorded in PERSYVE Q using an electronic diary. Patients will report spontaneously but will also be prompted via SMS at three occasions: after 3, 5, and 7 weeks of dosing.

^j^ Details specified in a laboratory manual.

^k^ Adverse events recorded by clinical staff at visits 2 and 3 in each treatment period (observations, open questions, and reporting of symptoms spontaneously mentioned by patients). PERSYVE Q completed before the open AE question.

^l^ At visit 3 in each treatment period patients will fill out a treatment preference rating Q consisting of five questions covering willingness to pay, treatment preference, safety, and motivation.

^m^ 24-h BP/ECG at the end of each treatment period, between visits 3 and 4. Applied at visit 3 and removed the same time at visit 4.

^n^ 24-h BP/ECG also at the end of placebo run-in to get a baseline assessment.

^o^ Overall patient preference rating and prescription of preferred study drug for continuous use.

Q = questionnaire.

The first patient was randomized in the first quarter of 2017; last patient out is expected in the fourth quarter of 2021.

### Study visits and periods

*Visit 1.* The screening visit (visit 1) will occur at least two weeks before randomization (visit 2). Written informed consent is obtained, eligibility criteria (described in Table 3) checked, and assessments are made of weight, height, waist, hip circumference; a physical examination is carried out, concomitant medication is noted, office blood pressure is measured, and blood is sampled. The patient fills out two questionnaires covering medical history and beliefs about medicine. All questionnaires in the study are summarized in Table 4.

**Table 3.** Inclusion and exclusion criteria.

Inclusion criteria
Male or female aged ≥40 years and ≤75 years
Previously diagnosed with hypertension with systolic blood pressure between 140 and 159 mmHg within 5 years prior to the start of the trial
Pharmacologically untreated or using blood pressure-lowering monotherapy at visit 1. No blood pressure-lowering medication taken during the placebo run-in period
Office systolic blood pressure between 140 and 179 mmHg and diastolic blood pressure at or below 109 mmHg at visit 2
Patients must give informed consent to participate in the study
Exclusion criteria
Medical history, clinical signs, or laboratory results indicating secondary hypertension, including primary aldosteronism or renal artery stenosis
Evidence of serious hematological, respiratory immunological, renal, hepatic, gastrointestinal, endocrinological, metabolic, neurologic, malignant, psychiatric, or other diseases as revealed by medical history, physical examination, and/or laboratory assessments
Active gout
Previous or present arterial occlusive diseases such as myocardial infarction (MI), stroke or acute arterial insufficiency (unstable angina pectoris or transient ischemic attacks [TIA]), or heart failure (NYHA class III or IV, or left ventricular systolic dysfunction irrespectively of function class)
Moderate or severe aortic or mitral insufficiency
Renal failure, including hemodialysis or kidney transplant(s)
Atrial fibrillation in need of rate control
Symptomatic hypotension, defined as weakness or syncope upon rising to an erect position associated with a decrease in systolic blood pressure
Diabetes requiring insulin or oral glucose-lowering drugs
Any history of serious abnormal drug reaction to active or inactive compounds in the study drugs, including angioedema
Any condition associated with poor compliance including alcoholism or drug dependence
Patients who will not comply with the study protocol as judged by the investigator
Women who are pregnant or lactating or not using appropriate contraception for at least 3 months prior to visit 1. Acceptable contraceptive methods are: combined (estrogen and progesterone containing) hormonal contraception associated with inhibition of ovulation (oral, intravaginal, transdermal), progesterone-only hormonal contraception associated with inhibition of ovulation (oral, injectable, implantable), intrauterine device, intrauterine hormone-releasing system, bilateral tubal occlusion, and vasectomized partner
Continuous use of concomitant medication that can interfere with study medication, i.e. digitalis glucosides, sotalol, cholestyramine, colestipol, NSAID, lithium, carbamazepine, CYP3A4-inhibitors, CYP3A4-inducers, dantrolene, diuretics, aliskiren, gold, sympathomimetics, tricyclic antidepressants, antipsychotics, anaesthetics, and potassium supplements
Clinical laboratory assessment outside normal range at visit 1 and judged clinically significant by the investigator
Previous randomization in present study

**Table 4.** Questionnaires.

At visit 1 the patients complete a questionnaire covering demographic items, medical history, smoking, and physical activity.
At visit 1 the patients complete a ‘Beliefs about medicine’ questionnaire.
At visit 2 after placebo run-in and at visit 3 after each treatment period, patients fill out a modified section 2.1 of the ‘PERSYVE’ questionnaire on paper in the clinic. Patients are also prompted via SMS to fill out the questionnaire at home at specific time points—at week 3, week 5, and week 7 of each treatment period, on paper or using an electronic diary (provided by Symptoms Europe AB, filled out via smartphone).
At visit 3 in each treatment period patients will fill out a ‘Treatment preference rating questionnaire’ consisting of five questions covering willingness to pay, treatment preference, safety, and motivation.
At visit 5 patients fill out an ‘Overall treatment preference rating’.

Written lifestyle advice (physical activity, weight loss, dietary changes, stress management, etc.) in accordance with national guidelines (SBU 2007) is provided to all participants.

*Placebo run-in period.* After the screening visit, the patient stops taking any concomitant blood pressure-lowering drugs and starts on a placebo run-in period of two weeks to establish an untreated baseline to be used for characterization of the study sample.

*Visit 2.* At visit 2, eligibility criteria are checked once again. Assessments made at this visit include fasting venous and capillary blood sampling, questionnaires, and pulse wave analysis, and 24-h blood pressure and ECG monitoring is initiated.

If all criteria are met, the patient is randomized and given the first study drug. The study drug is to be taken once daily.

The day after visit 2, the patient returns the 24-h blood pressure and ECG equipment to the clinic.

*Treatment period.* Between visits 2 and 3 patients are dosing study drug at home, and fill out the side effects questionnaire at home at specific time points.

*Visit 3.* At visit 3 of each treatment period (which occurs after 4–6 weeks on the target dose of the study drug, 7–9 weeks after the previous visit 3) the patient comes fasting to the clinic for blood sampling, body weight, concomitant medication, and adverse events (AE) checks, office BP measurement, and initiation of 24-h blood pressure and ECG measurement. The patient fills out two paper questionnaires: the side effect questionnaire (identical to the questionnaire for home use) and a treatment preference rating questionnaire. The patient returns all unused study drug (ensuring that they have full coverage of study drug until the completion of visit 4) and receives the next treatment kit, which starts with a 7-day placebo wash-out period before the next active drug treatment period starts.

In rare cases, if a patient due to unbearable side effects of a specific treatment so wishes, bailout visits 3 and 4 can be carried out and the treatment period terminated prematurely so that the next treatment period can begin.

*Visit 4.* At visit 4, the day after visit 3, the 24-h blood pressure and ECG measurement ends and the patient returns the equipment to the clinic. The treatment periods and visits 3 and 4 are repeated for a total of 6 times.

*Visit 5.* Visit 5 occurs 1–2 weeks after the sixth and last visit 4. At visit 5, the patient is asked by an independent physician to do an overall treatment preference rating including all treatment periods, based on a summary of the patient’s own reported side effects, treatment preference ratings for all treatment periods, and ambulatory daytime blood pressures. All data in the summary have been validated by data management personnel, signed by the PI and locked before being presented to the patient. As a final step, the independent physician breaks the randomization code for each completed patient, and prescribes the drug preferred by the patient for continuous use.

## Statistical considerations

### Analysis population

Intention-to-treat analyses will be used for all side effects, including all patients taking at least one dose of the study drug in a new treatment period. As-randomized analyses will be used for blood pressure endpoints, including all patients having had a treatment adherence of more than 90% during that treatment period.

### Primary analyses: individual variation in treatment differences

The primary analysis of individual variation in treatment differences in visit 4 daytime systolic blood pressure will be performed using a random effects model with correlated random intercept and treatment contrasts, allowing correlation between all random effects, and with treatment period as a fixed effect. All available periods with >90% treatment adherence from all randomized patients will be used. The global hypothesis—that there is a contribution from patient-specific differences in relative treatment response to the variation in on-treatment blood pressure in the analysis set—will be tested using a likelihood ratio test based on the maximum-likelihood fit of that model compared to a model with random intercept (only) and treatment contrasts and treatment period as fixed effects. Since variance parameters must be non-negative, the null hypothesis lies on the border of the parameter space of the larger model. Hence the usual assumption of chi-square distribution in the likelihood ratio test is not valid, and the test statistic will be compared to a reference distribution obtained by parametric bootstrapping to obtain a *p* values. *P* < 0.05 will be considered statistically significant.

Appropriate methods will be used to present estimates of treatment contrast variances, covariances, and residual variance. Homoscedasticity of the residual variance will be investigated. A detailed statistical analysis plan for pre-defined analyses will be finalized before unblinding. Problems with model convergence or other changes to the analysis will be recorded in a statistical report.

For each pair of treatment regimens (say *X* and *Y*), descriptive plots and tables will be produced based on the patients that had repeated periods with both treatment *X* and *Y*. A pre-defined pairing of the periods for each patient, into what amounts to two cross-over trials in the same patient, will be used to produce two independent measurements of the treatment contrast *X–Y* within each patient.

Corresponding analyses will be performed for other blood pressure measurements. Similar analyses, adapted for type and distribution of the variable, will be performed for visit 3 measurements of other outcome variables, as appropriate. Pre-defined analyses will be described in the SAP. No formal adjustment for multiplicity will be performed in the primary analyses.

### Secondary analyses: exploratory biomarker analyses

If the primary analyses indicate that there may be a substantial between-person variation in effect of treatment on blood pressure, exploratory analyses will be conducted developing prediction models for individual effects on blood pressure. Prediction models for side effects will be pursued irrespectively of results of the primary models. Models will be developed using DNA, RNA, and other biomarkers to predict individual treatment effects on blood pressure and side effects. The Institute of Medicine guidelines for development and validation of omics-based tests will be used (15). Because of the limited sample size, the cross-validation approach will be prioritized over training/test set sample division approaches. Similar models as in the primary analyses will be used, typically involving mixed models with fixed effects for most of the independent variables, and random effects across patients and other structural elements. Significance level correction for multiple testing approaches such as 5% FDR will be applied.

In further exploratory analyses we will use instrumental variable regression techniques combining genomic DNA with DNA methylation/RNA/protein/metabolite data (Mendelian randomization) to address the causal role of the identified biomarkers for blood pressure response and side effects, which is unimportant for prediction purposes, but key for further investigation of new druggable targets.

### Determination of sample size

Statistical power for detecting between-patient variation in treatment effects was simulated using R v. 3.2.2 (R Foundation for Statistical Computing, Vienna, Austria), and packages lme4 v. 1.1–10 (16) and pbkrtest v. 0.4–4 (17). Altogether 500 trials were simulated for each alternative hypothesis, and for each simulated trial a *p* values was computed using 1000 bootstrap iterations for the reference distribution of the test statistic (Table 5). *P* < 0.05 was considered a significant outcome. The trials were simulated without period effects, and the analysis model did not include period as a factor. The validity of the method was investigated by simulating power under the null hypothesis. The on-treatment standard deviation of between- and within-patient variation in systolic blood pressure was assumed to be 14 and 12 mmHg, respectively, based on repeated measurements about 1 month apart in 21 patients.

**Table 5.** Summary of power simulation.

Simulation model (mmHg): $SBP_{i}=\text{Intercep}t_{i}+Ind_{\text{TreatA}}\times \text{Treat}A_{i}+Ind_{\text{TreatB}}\times \text{Treat}B_{i}+Ind_{\text{TreatC}}\times \text{Treat}C_{i}+Ind_{\text{TreatD}}\times \text{Treat}D_{i}+\epsilon_{i,\;\text{period}}$ Between-patient variation assumption: $\text{Intercep}t_{i}∼\;N\left(0,14\right)$ Within-patient variation assumption: $\epsilon_{i,\text{period}}∼N(0,12)$				
	
Null hypothesis with *N* = 240 (for validation purpose, should be 5%) $\text{Treat}A_{i}=\;\text{Treat}B_{i}=\text{Treat}C_{i}=\;\text{Treat}D_{i}=0$				
5.2%				
	
Normally distributed individual treatment effects with SD = *x* mmHg $\text{Treat}A_{i}∼\;N\left(0,a\right),\;\text{Treat}B_{i}∼\;N\left(0,b\right),\;\text{Treat}C_{i}∼\;N\left(0,c\right),\;\text{Treat}D_{i}∼\;N\left(0,d\right)$				
*N* = 240				
		x=5	x=7.5	x=10
a=b=c=0,d=x		29%	82%	>99%
a=b=0,c=d=x		46%	97%	>99%
a=0,b=c=d=x		57%	>99%	>99%
a=b=c=d=x		64%	>99%	>99%
				
A fraction *p* of patients have *x* mmHg uniformly higher BP on treatment D (note that $SD\left(\text{Treat}D_{i}\right)=\sqrt{p\times \left(1-p\right)}\times x$, maximum SD=*x/2* at 50% fraction) $\text{Treat}D_{i}∼\;B(1,p)\times x$				
*N* = 240				
	x=10		x=20	x=30
p=20%	12%		90%	>99%
p=50%	27%		>99%	>99%

Two scenarios were simulated. In the first, normally distributed uncorrelated individual treatment effects were added to between one and four of the treatment arms, with a standard deviation of 5, 7.5, or 10 mmHg. With *n* = 240 patients, the trial has a power of 82% for detecting individual treatment effects if only one treatment arm differs from the others by having an individual variation in effect with SD =7.5 mmHg, and a very high power if two or three treatments have patient-specific effects on that level.

The second scenario is intended to describe a simplified on/off switch for effect. We assume that for one of the treatments 20%, or 50%, of the patients experience an effect that is 10, 20, or 30 mmHg higher (or lower) than the remaining patients. The trial has 90% power to show if a subgroup of 20% of patients has an effect that differs by 20 mmHg on one of the treatments. In terms of SD of treatment effects, the power is similar to the simulations under normal distribution (the SD is the subgroup effect times 0.5 for the 50% case and times 0.4 for the 20% case).

## Study conduct

### Permissions

The study was approved by the Ethics Committee of Uppsala University (Dnr 2016/135) and the Swedish Medical Products Agency (Dnr 5.1–2016-25102; EudraCT 2015–003049-24). The study follows a protocol pre-registered at ClinicalTrials.gov with the identifier NCT02774460. The study is conducted in accordance with the protocol, applicable regulatory requirements, GCP, and the ethical principles of the Declaration of Helsinki as adopted by the 18th World Medical Assembly in Helsinki, Finland, in 1964 and subsequent versions. Written informed consent will be obtained from all patients prior to enrolment in the study.

### Randomization and blinding

The study will be patient-, investigator-, study nurse-, UCR study team-, and sponsor-blinded. Clinical examinations, interpretations of 24-h blood pressure and ECG measurements, and assessments of side effects, treatment preferences, and other questionnaires will be performed by persons unaware of the treatment allocation.

The order of the different active treatments is randomized for each patient. The patients are assigned consecutive randomization numbers according to their inclusion order, and clinical staff will record the numbers being used. The randomization list is prepared at Uppsala Clinical Research Center (UCR).

The investigational products are manufactured and packaged in inconspicuous blister packaging and labelled by Apotek Produktion och Laboratorier AB (APL) in accordance with good manufacturing practice (GMP) and local regulatory guidelines.

### Data management and monitoring

Electronic case report forms (eCRF) are used for all data collection, in accordance with a pre-specified data management plan. In accordance with GCP principles, monitoring of the study is arranged by the sponsor, and carried out by UCR, in accordance with a pre-specified monitoring plan.

This study uses two study databases, database #1 for the majority of the study, and database #2 for visit 5. On an individual patient level, after the last visit 4, database #1 is validated by data management personnel, signed by the PI, and locked, before being presented to the patient by an independent physician. After each patient has provided a new overall treatment preference rating, the independent physician signs the eCRF, breaks the randomization code for that patient, and enters the eCRF data into database #2. Upon study completion databases #1 and #2 are merged.

## Perspective

In the PHYSIC trial, we use the only design that can study the potential for precision medicine for blood pressure-lowering treatment (12). The crucial disentangling of all variance components has not been possible in previous studies of this research question (8–10). We reproduce a previous trial (9), removing several important sources of bias, increasing the sample size 5-fold, and using ambulatory blood pressure measurements that further increase power. The concept of n-of-1 trials to guide treatment decisions has been advocated (18), and it is akin to the PHYSIC study design but on an individual level (19). Because the evaluation of blood pressure effects should not be done until after a month on the target dose, n-of-1 trials of four classes of blood pressure-lowering drugs would be prohibitively long if all treatment contrasts were to be repeated in an individual patient. Hence the appeal of a biomarker panel to guide treatment choice. Importantly, our study design has the power to rule out any potential for biomarker-based precision hypertension care, in which case research resources are better spent elsewhere. It can also inform on the sensibleness of n-of-1 trials in hypertension, or, simply put, the usefulness of trying another drug if a patient experiences side effects or questionable blood pressure effects of the first drug.
